# Hybrid femoral-to-above-knee popliteal bypass with popliteal relining for complex femoropopliteal occlusive disease

**DOI:** 10.1016/j.jvscit.2026.102353

**Published:** 2026-06-09

**Authors:** Qinghua Pu, Logan Stone, Sirui Pu, Mahmoud Almadani, Alexander Shiferson, Albertina Sebastian, Robert Rhee

**Affiliations:** a Division of Vascular Surgery, Maimonides Medical Center, Brooklyn, NY; b Department of Computational Biology, College of Agriculture and Life Sciences, Cornell University, Ithaca, NY

**Keywords:** Chronic limb-threatening ischemia, Femoropopliteal occlusive disease, Hybrid revascularization, Below-knee popliteal bypass, Covered stent-graft, Prosthetic bypass

## Abstract

**Objective:**

Complex femoropopliteal occlusive disease (CFPOD) involving superficial femoral artery occlusion with concomitant above-knee (AK) popliteal disease is commonly treated with femoral-to-below-knee popliteal bypass. However, outcomes are limited when autologous vein is unavailable and prosthetic conduits are used for infrageniculate targets. We explored a hybrid femoral-to-above-knee popliteal bypass (HYB) as an alternative treatment for CFPOD.

**Methods:**

HYB was performed in three patients by combining endovascular reconstruction of the popliteal artery using balloon angioplasty and covered stent graft relining to create a functional AK outflow segment with a femoral-to-AK popliteal bypass. The technical details of the procedure were described and illustrated. Operative data, clinical outcomes, and midterm graft patency were recorded.

**Results:**

Three patients with chronic limb-threatening ischemia and a history of failed interventions with no suitable vein conduit underwent HYB. Operative time was approximately 3 hours, and mean blood loss was 300 mL. Technical success was achieved in all cases, with no perioperative morbidity or mortality. All patients achieved limb salvage, sustained symptom relief, and maintained graft patency for at least 2 years, including one patient with follow-up beyond 3 years.

**Conclusions:**

HYB with popliteal relining may offer a durable alternative to prosthetic femoral-to-below-knee popliteal bypass in selected patients with CFPOD and limited conduit options. Larger comparative studies are needed to define long-term outcomes and optimal patient selection.

**Article Highlights**

Hybrid femoral-to-above-knee popliteal bypass combines prothetic bypass with balloon angioplasty and covered stent-graft relining of the diseased popliteal artery to create a functional above-knee outflow target. In three patients with chronic limb-threatening ischemia and no suitable autologous vein conduit, the technique achieved technical success, limb salvage, and sustained patency for at least 2 years.

Peripheral artery disease (PAD) is a major cause of functional impairment and limb loss. Patients with chronic limb-threatening ischemia (CLTI) face substantial risks of amputation and mortality, and contemporary guidelines emphasize individualized revascularization to achieve durable limb-based patency.^1^ Further evidence highlights the importance of conduit selection and revascularization strategy in determining clinical outcomes in CLTI.^2^

Femoropopliteal bypass remains a key revascularization strategy for long superficial femoral artery (SFA) occlusions and complex disease patterns. However, outcomes depend heavily on conduit and target level. In aggregate data, autologous vein demonstrates superior patency to prosthetic grafts; prosthetic performance is especially limited when distal targets are infrageniculate.^3^, ^4^, ^5^ When complex femoropopliteal occlusive disease (CFPOD) includes AK popliteal stenosis/occlusion, surgeons may default to femoral-to-below-knee popliteal (Fem-BK Pop) bypass to reach healthier outflow. Yet in patients without usable vein, prosthetic Fem-BK Pop bypass or cryopreserved conduit may be associated with inferior durability.^6^^,^^7^

Covered stent grafts (eg, heparin-bonded Viabahn) have shown clinically meaningful patency in complex femoropopliteal lesions in randomized studies.^8^^,^^9^ These data support a hybrid concept: endovascularly “reconstruct” the AK popliteal artery to create a suitable above-knee (AK) outflow segment, then perform a femoral-to-above-knee popliteal (Fem-AK Pop) bypass with a prosthetic graft.

We describe a reproducible hybrid technique using popliteal relining to enable AK bypass and present early clinical outcomes.

## Technique: hybrid femoral-to-above-knee popliteal bypass with popliteal relining

### Patient selection

Patients were considered for hybrid femoropopliteal bypass when they had CFPOD, defined as femoral artery occlusive disease extending into the popliteal artery. Candidates also had at least one feature making conventional bypass less favorable, including absence of suitable autologous vein, severe comorbidities limiting tolerance for a prolonged bypass procedure, or morbid obesity with body mass index > 40 kg/m^2^. Patients with acute arterial occlusion or thrombosis, or arterial occlusion unrelated to peripheral arterial disease, were excluded.

### Setting and exposure

Procedures were performed in a hybrid operating suite under general anesthesia. Separate groin and medial thigh incisions were made to expose the common femoral artery and the AK popliteal artery. A ringed Propaten graft (W. L. Gore & Associates) was tunneled between the incisions. The distal graft is trimmed to leave a short nonringed segment.

### Direct popliteal access and diagnostic angiography

**Fig 1:**
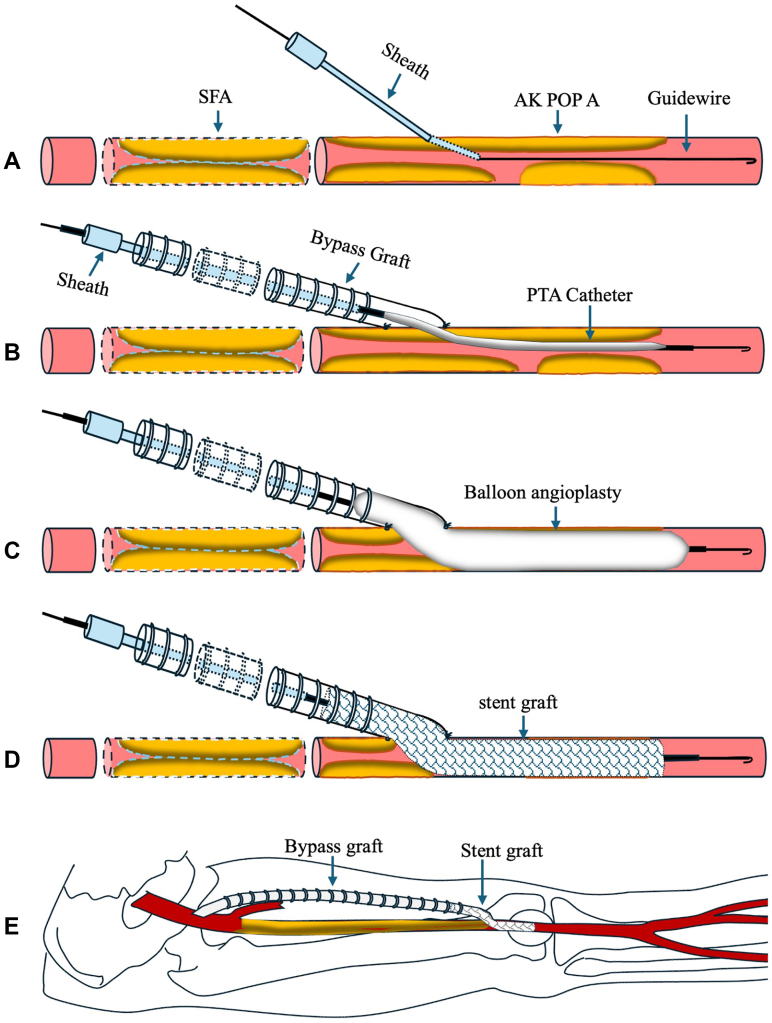
Schematic illustration of hybrid femoral-above-knee popliteal (Fem-AK Pop) bypass. **A,** Open exposure of a stenotic above-knee (*AK*) popliteal artery with direct arterial access using a sheath and guidewire. **B,** Introduction of a long sheath through a pretunneled bypass graft, with backloading of the guidewire and advancement of the sheath into the popliteal artery, followed by placement of a percutaneous transluminal angioplasty (*PTA*) catheter. **C,** Balloon angioplasty of the popliteal artery with dilation of the access site. **D,** Relining of the popliteal artery with a covered stent graft extending into the bypass graft to create a sutureless distal anastomosis. **E,** Completion of the proximal anastomosis, establishing a hybrid Fem-AK Pop bypass.

Under direct visualization, the AK popliteal artery is accessed using a micropuncture needle and wire. If the AK popliteal artery is occluded, a limited arteriotomy or partial transection may be performed to facilitate sheath entry. This approach provides direct visualization of the arterial lumen, enabling more reliable cannulation of the occluded popliteal segment, and facilitating wire crossing and stent deployment. A 5F sheath is then placed, and antegrade angiography is performed to delineate the popliteal and tibial runoff. A 0.018-inch V-18 ControlWire guidewire (Boston Scientific) is advanced into a tibial vessel (Fig 1, *A*).

### Sheath-through-graft maneuver and popliteal artery angioplasty

A long introducer sheath (6-7F, ∼55 cm) is placed through the Propaten graft. After removal of the 5F sheath, the 0.018-inch wire is backed loaded to the long introducer sheath, which was subsequently advanced into the popliteal artery over the wire (Fig 1, *B*). The distal end of the polytetrafluoroethylene (PTFE) graft is secured and aligned with the native AK popliteal artery at the access site using four interrupted 5-0 polypropylene sutures placed circumferentially. Balloon angioplasty is then performed to treat popliteal stenosis/occlusion and expand the access zone (balloon diameter typically matched to graft size) (Fig 1, *C*).

### Stent-graft relining and distal anastomosis

A Viabahn stent-graft (W. L. Gore & Associates), sized to match the Propaten graft at a 1:1 nominal diameter, is deployed to reline the diseased popliteal segment, with ≥5 cm overlap into the Propaten graft to create a sutureless anastomosis between the bypass graft and the AK popliteal artery. The interface is then balloon-molded to optimize seal and luminal transition (Fig 1, *D*).

This 1:1 sizing strategy was used because the native popliteal arteries in our series were often small, frequently <5 mm, whereas the smallest practical prosthetic graft was 6 mm. Although the Viabahn IFU recommends approximately 10% oversizing, sizing based on the graft could result in excessive oversizing within the native popliteal artery. As the Viabahn outer diameter exceeds its nominal inner diameter, 1:1 nominal sizing provided adequate apposition within the PTFE graft while minimizing excessive radial force in the diseased popliteal artery, particularly when ≥5 cm graft overlap was achieved.

### Proximal anastomosis and completion imaging

A proximal end-to-side anastomosis is created to the common femoral artery to accomplish the hybrid femoral-to-above-knee popliteal bypass (HYB) (Fig 1, *E*). Completion angiography confirms graft patency and runoff.

### Postoperative antithrombotic strategy

Postoperative antithrombotic therapy was individualized according to each patient's thrombotic risk profile. In patients with PTFE bypass and one or more high-risk features—single-vessel runoff, earlier failed bypass, known hypercoagulability, or previous thromboembolic events—we favored therapeutic anticoagulation in combination with single antiplatelet therapy. In patients without these high-risk features, dual antiplatelet therapy was typically used after hybrid bypass. In this case series, patients received either antiplatelet therapy plus anticoagulation or dual-pathway therapy according to these criteria.

## Case presentations

### Case 1

A 60-year-old woman presented with 8 months of left lower extremity rest pain and multiple ischemic ulcers. She had undergone multiple earlier femoropopliteal interventions, including angioplasty, atherectomy, and stenting. She had a 40 pack-year smoking history and had quit 9 months earlier. Her medical history was notable for ischemic stroke 3 months previously, ovarian cancer surgery 2 months earlier with planned chemotherapy, and previous deep vein thrombosis on anticoagulation.

**Fig 2:**
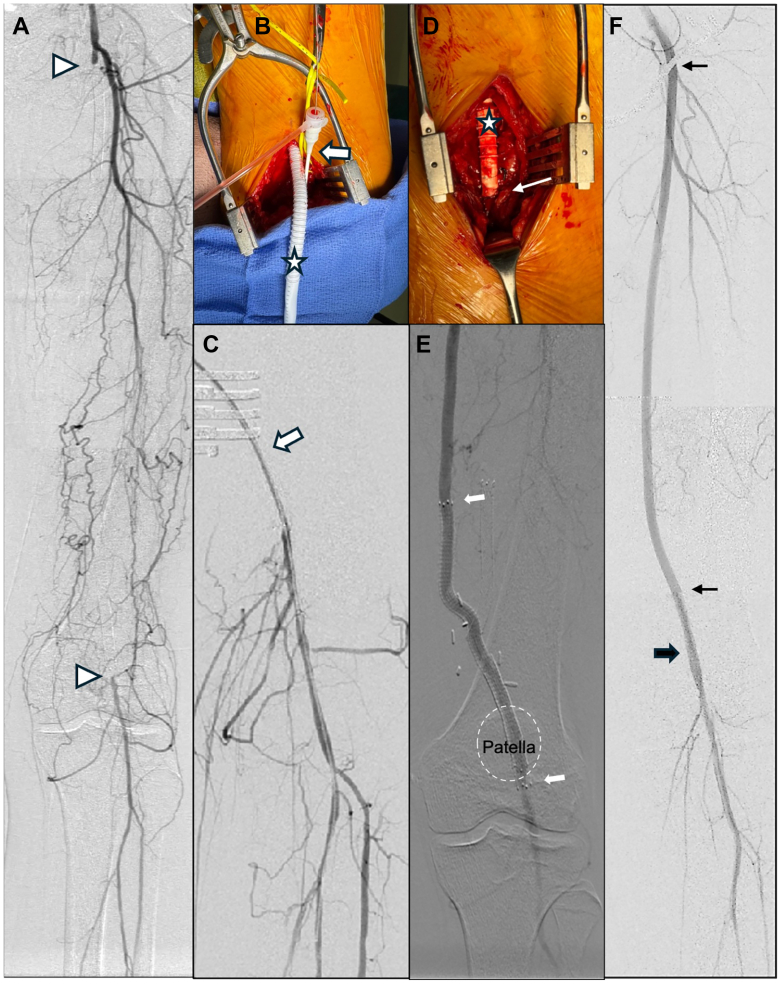
Hybrid femoral-above-knee (AK) popliteal bypass with endovascular reconstruction of AK popliteal artery (Case 1). **A,** Preoperative angiography demonstrating chronic total occlusion of the right superficial femoral artery and AK popliteal artery (*arrowheads*). **B,** Open exposure of the AK popliteal artery with direct arterial access using a 5F sheath (*arrow*); the bypass graft is pretunneled (*asterisk*). **C,** Angiography confirming successful intraluminal cannulation of the AK popliteal artery with guidewire and sheath (*arrow*). **D,** Creation of a sutureless anastomosis using a covered stent bridging the AK popliteal artery and the bypass graft (*asterisk* indicates stent graft; *arrow* indicates graft). **E,** Popliteal artery relining after angioplasty, with a Viabahn stent-graft extending from the P2 segment into the polytetrafluoroethylene (PTFE) graft (*arrows*). **F,** Completion angiography demonstrating a patent hybrid femoral-popliteal bypass (*thin arrows*) and reconstructed popliteal artery (*thick arrow*).

Duplex ultrasound study demonstrated occlusion of the left SFA and AK popliteal artery, with distal tibial disease and a nondetectable ankle-brachial index. Angiography confirmed SFA occlusion, an occluded AK popliteal stent, a patent below-knee popliteal artery, and segmental tibial occlusions with robust collateralization (Fig 2, *A*). Endovascular recanalization was unsuccessful. Vein mapping demonstrated bilateral great saphenous veins <2 mm.

After multidisciplinary coordination, the patient completed 3 months of chemotherapy and returned with persistent CLTI and toe gangrene. A hybrid Fem-AK Pop bypass was performed. The AK popliteal artery was directly accessed through a partially transected arterial segment and earlier stent (Fig 2, *B*). Angiography confirmed significant luminal disease (Fig 2, *C*). Following balloon angioplasty, a 6-mm × 15-cm Viabahn stent graft was deployed into the P2 segment of the popliteal artery to reline the diseased vessel and bridge into the ringed Propaten graft, creating a continuous graft-artery interface (Fig 2, *D* and *E*). Restoration of popliteal flow was confirmed angiographically (Fig 2, *E*).

After completion of the proximal anastomosis, final angiography demonstrated a widely patent hybrid bypass with brisk tibial runoff (Fig 2, *F*). Operative time was 207 minutes with an estimated blood loss of 200 mL. The patient was discharged on postoperative day 2 on clopidogrel 75 mg daily and continuation of home anticoagulation (aspirin discontinued). The ankle-brachial index improved to 1.05, rest pain resolved immediately. She received debridement of the toe gangrene and 12 sessions of hyperbaric oxygen therapy. The gangrene completely healed by 11 months. At 12 months, she underwent angiography for high-grade tibioperoneal trunk stenosis, which was treated with drug-coated balloon angioplasty. The bypass remained patent 30 months postoperatively.

### Case 2

A 78-year-old woman presented with severe right lower extremity rest pain and a 7-cm × 5-cm dorsal foot ulcer with exposed extensor tendons for 2 weeks. Her comorbidities included hypertension, insulin-dependent diabetes mellitus, hyperlipidemia, anemia, hypoalbuminemia, and PAD. The initial angiogram demonstrated chronic total occlusion of the right SFA, with multiple stenoses involving the popliteal artery. Complete endovascular reconstruction was attempted initially but was unsuccessful. She underwent a conventional right Fem-BK Pop bypass with a Propaten graft 10 weeks earlier for rest pain and tissue loss. Although her symptoms initially improved, she developed recurrent rest pain and worsening of right foot ulcer over the preceding 2 weeks, and duplex ultrasound study demonstrated graft occlusion.

**Fig 3:**
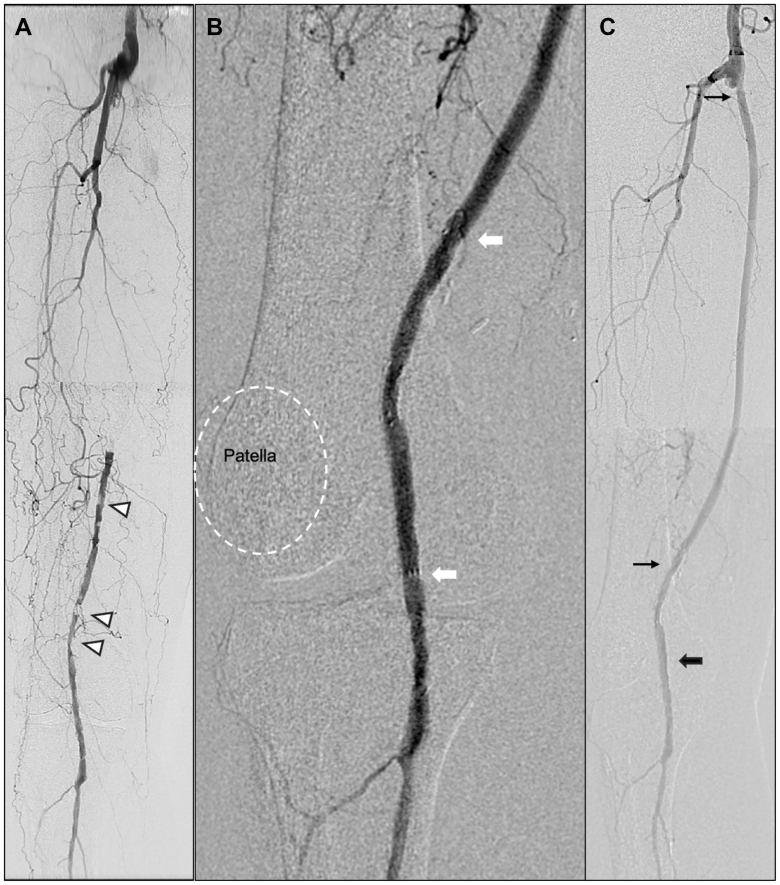
Pre- and postprocedural angiography of hybrid femoral-above-knee popliteal bypass (Case 2). **A,** Preoperative angiography demonstrating chronic total occlusion of the left superficial artery (*arrows*) with multiple high-grade stenoses in the above-knee popliteal artery (*arrowheads*). **B,** Popliteal artery relining after angioplasty, with a Viabahn stent graft extending from the P2 segment into the polytetrafluoroethylene (PTFE) graft (*arrows*). **C,** Completion angiography demonstrating a patent hybrid femoral-popliteal bypass (*thin arrows*) and reconstructed popliteal artery (*thick arrow*).

Repeat angiography revealed chronic occlusion of the right SFA, multiple focal stenoses of the AK popliteal artery, and a patent below-knee popliteal artery with single-vessel peroneal runoff (Fig 3, *A*).

Given early failure of the prosthetic Fem-BK Pop bypass and lack of suitable vein conduit, a hybrid Fem-AK Pop bypass was performed. The AK popliteal artery was accessed and treated with balloon angioplasty. A 6 × 150-mm Viabahn stent graft was deployed into the P2 segment to reline the diseased popliteal artery and bridge into a 6-mm ringed Propaten graft (Fig 3, *B*). A proximal end-to-side anastomosis was then created to the common femoral artery. Completion angiography demonstrated a patent hybrid bypass with brisk peroneal runoff (Fig 3, *C*).

Operative time was 182 minutes with an estimated blood loss of 400 mL. The patient was discharged to subacute rehabilitation on postoperative day 3 with apixaban 5 mg twice daily and clopidogrel 75 mg daily. The foot ulcer was complicated by infection and chronic osteomyelitis involving the first phalanx. The wound ultimately healed at 18 months after multiple debridements and prolonged antibiotic therapy. The bypass remained patent on clinical examination and duplex surveillance through 3 years, after which the patient was lost to follow-up.

### Case 3

A 70-year-old man presented with worsening left lower extremity rest pain, numbness, and a toe ulcer. He had a history of smoking (10 cigarettes daily for 30 years) and extensive PAD, including earlier iliac stenting and bilateral SFA stenting, followed by two right femoral-distal bypasses and a right transmetatarsal amputation.

**Fig 4:**
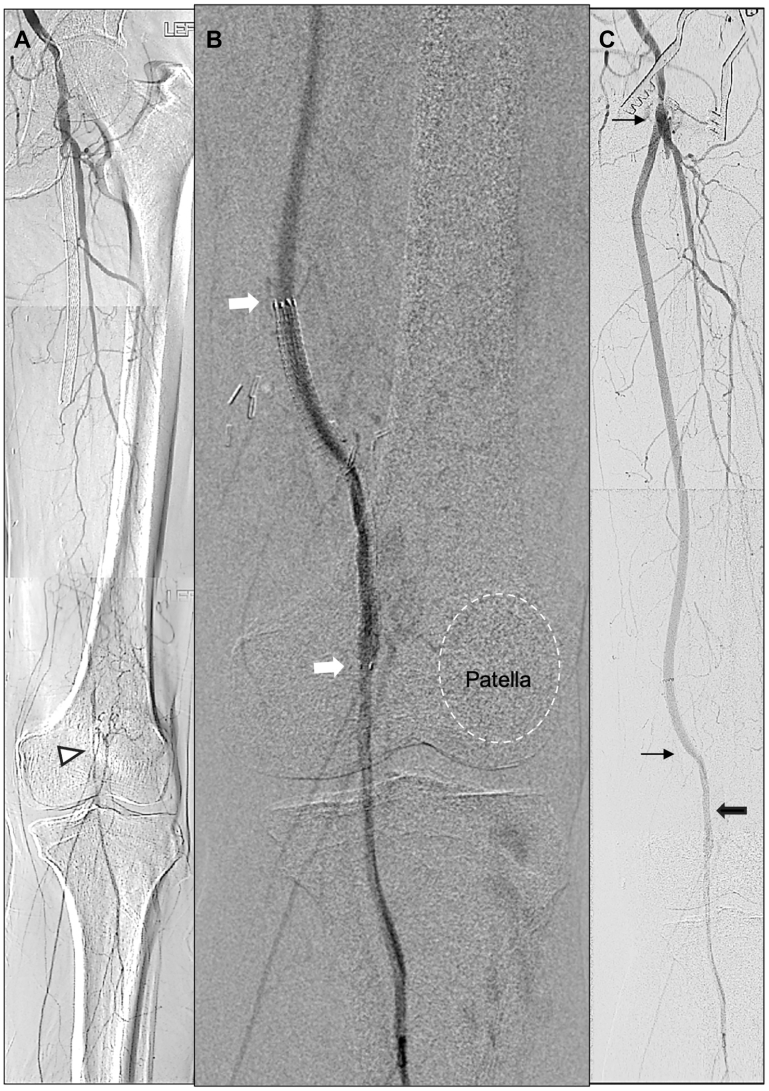
Pre- and postprocedural angiography of hybrid femoral-above-knee-popliteal bypass (Case 3). **A,** Preoperative angiography demonstrating chronic occlusion of the left superficial femoral artery (*arrows*) and a small-caliber above-knee popliteal artery (2-3 mm; *arrowhead*). **B,** Popliteal artery relining after angioplasty, with a Viabahn stent graft extending from the P2 segment into the polytetrafluoroethylene (PTFE) graft (*arrows*). **C,** Completion angiography demonstrating a patent hybrid femoral-popliteal bypass (*thin arrows*) and reconstructed popliteal artery (*thick arrow*).

On presentation, the left foot was cold with dependent rubor and first-toe ulceration. Angiography revealed chronic total occlusion of the left SFA, a diffusely small-caliber (2∼3 mm) popliteal artery and tibioperoneal trunk, a 2-cm AK popliteal occlusion, and single-vessel peroneal runoff (Fig 4, *A*). Endovascular therapy was unsuccessful. Due to lack of usable vein following earlier contralateral bypasses, a hybrid Fem-AK Pop bypass was performed.

The AK popliteal artery was exposed and partially transected to facilitate sheath placement and wire crossing. Balloon angioplasty was performed to expand the popliteal artery and tibioperoneal trunk to 4 to 5 mm. A 6-mm × 10-cm Viabahn stent graft was then deployed to reline the AK popliteal artery to P2 segment and extend into a 6-mm Propaten graft (Fig 4, *B*). Completion angiography demonstrated a patent graft with restoration of popliteal flow and satisfactory runoff (Fig 4, *C*).

Operative time was 179 minutes with an estimated blood loss of 300 mL. The patient was discharged on postoperative day 3 with resolution of rest pain and improvement in toe ulceration. The bypass remained patent 28 months after surgery.

## Discussion

This report highlights a hybrid approach for CFPOD in which AK popliteal stenosis or occlusion precludes a conventional Fem-AK bypass target and absent autologous vein limits durable options. In such patients, prosthetic Fem-BK Pop bypass or cryopreserved conduits may be considered; however, both strategies have important durability limitations. Prosthetic Fem-BK Pop bypass has demonstrated inferior long-term primary patency compared with AK prosthetic bypass, with reported 5-year primary patency rates of approximately 40% to 44% for below-knee PTFE grafts.^6^ Cryopreserved vein grafts have similarly limited durability, with reported primary patency rates of approximately 37% at 1 year and 24% at 3 years.^7^ The hemodynamic and durability advantages of AK reconstruction are well recognized, with superior outcomes compared with infrageniculate bypass, particularly when prosthetic conduits are used.^3^, ^4^, ^5^ When vein is unavailable, heparin-bonded ePTFE may improve prosthetic durability but still does not match autologous vein performance.^4^^,^^5^

Our strategy leverages endovascular “target creation” or “target optimization” by relining the diseased AK popliteal artery with a covered stent graft, thereby converting a poor quality or unusable target into a functional outflow vessel. This approach aligns with contemporary CLTI principles emphasizing restoration of a durable target artery path to achieve limb-based patency.^10^ Covered stent grafts have demonstrated favorable patency in complex femoropopliteal disease. The Viabahn Endoprosthesis with PROPATEN Bioactive Surface (VIA) Versus Bare Nitinol Stent in the Treatment of Long Lesions in Superficial Femoral Artery Occlusive Disease (VIASTAR) trial reported patency benefits for heparin-bonded covered stents compared with bare-metal stents in long lesions.^8^ Similarly, the Viabahn Versus Bare Nitinol Stent in the Treatment of Long Lesion (≥ 8 cm) Superficial Femoral Artery Occlusive Disease (VIBRANT) tiral demonstrated comparable secondary patency between bare stents and stent grafts, although differences were observed in primary-assisted patency and fracture rates.^9^ These data support the concept that a covered stent graft can provide a durable relined conduit within the popliteal segment, particularly when the alternative is an infrageniculate prosthetic bypass.

AK popliteal endarterectomy or other adjunctive techniques may also be considered to optimize the distal target in the selected patients. In our series, however, the AK popliteal arteries were diffusely diseased, often extending into the P2 segment or beyond. Open endarterectomy would therefore have been technically challenging in the supine position and might not have adequately treated the distal disease. Another option is full-length “Viabahn all-the-way” reconstruction. Although this strategy may be appropriate in selected patients, it requires successful crossing and relining of the entire SFA-popliteal occlusion and makes limb perfusion dependent on a long stent-graft construct. In our series, chronic SFA occlusion with diffuse AK popliteal disease made complete endovascular reconstruction less favorable or unsuccessful. Our hybrid approach instead bypasses the SFA occlusion to provide durable inflow, while using Viabahn selectively for popliteal relining, distal target optimization, and creation of a sutureless distal anastomosis.

All three patients in this case series had CLTI, as defined in contemporary guidelines, and anatomy/features expected to predict poor outcomes after prosthetic infrageniculate bypass, including limited runoff, earlier failed endovascular therapy, and absence of suitable vein. Case 1 shows applicability in the setting of occluded previous popliteal stents, where relining with a covered stent graft enabled a functional AK outflow. Case 2 illustrates a particularly high-risk scenario: early thrombosis of a previous prosthetic Fem-BK Pop bypass. Case 3 demonstrates feasibility in a small-caliber popliteal target with segmental occlusion.

Despite combining open and endovascular components, operative times were approximately 3 hours, with hospital length of stay 2 to 3 days, comparable to standard femoropopliteal bypass with synthetic graft. Limb salvage was achieved in all three cases. One patient required tibial reintervention at 12 months, consistent with the principle that limb-based patency in CLTI often requires surveillance and staged inflow-outflow optimization. Cases 1 and 2 had significant tissue loss and prolonged wound healing despite restoration of inline flow, reflecting the complexity of their disease. Wounds ultimately healed after adjunctive management, including hyperbaric oxygen therapy, debridement, and prolonged antibiotics. These cases highlight that wound healing requires not only revascularization but also multimodality management of infection, local wound care, nutrition, and systemic comorbidities.

The hybrid bypass technique should be distinguished from the Viabahn Open Revascularization TEChnique (VORTEC), which facilitates sutureless anastomosis using a telescoping stent graft.^11^, ^12^, ^13^ Although VORTEC has been applied in peripheral bypass, including limited reports on femoropopliteal reconstruction, published experience is confined to small case series demonstrating technical feasibility and short-term success, without robust long-term patency data specific to the femoropopliteal segment.^13^ Longer-term VORTEC outcomes are largely derived from renovisceral debranching procedures, where stent-graft patency has been reported to approach 80% to 85% at mid to long-term follow-up.^12^ However, these findings may not be generalizable to the popliteal artery given its unique biomechanical stresses and runoff dependence. Importantly, VORTEC primarily functions as a technical adjunct to simplify distal anastomosis and does not modify the quality of the outflow vessel, which remains a key determinant of bypass durability. In contrast, the present hybrid Fem-AK Pop bypass reconstructs the diseased popliteal artery and establishes a functional AK outflow target, representing a fundamental conceptual difference.

This report has several limitations, including its single-center design, small sample size, and lack of a control group. A comparative analysis evaluating long-term patency, limb salvage, complications, and cost-effectiveness relative to conventional Fem-BK Pop bypass strategies is currently being finalized, with results scheduled for presentation at the 2026 Vascular Annual Meeting.

## Conclusions

Hybrid femoral-to-AK popliteal bypass with AK popliteal angioplasty and covered stent-graft relining may offer a durable alternative for CFPOD when the AK popliteal artery is not an acceptable conventional bypass target and autologous vein is unavailable. In three CLTI cases, the technique achieved technical success, limb salvage, and sustained midterm patency with minimal perioperative morbidity. Further study is warranted to define patient selection and comparative effectiveness.

## Author Contributions

Conception and design: QP, RR

Analysis and interpretation: QP, LS, SP

Data collection: QP, LS, SP, MA, ASh, ASe

Writing the article: QP

Critical revision of the article: QP, LS, SP, MA, ASh, ASe, RR

Final approval of the article: QP, LS, SP, MA, ASh, ASe, RR

Statistical analysis: Not applicable

Obtained funding: Not applicable

Overall responsibility: QP

## Patient consent

Written informed consent for publication was obtained from all patients, and the study was approved by the institutional review board of Maimonides Medical Center.

## Declaration of generative AI and AI-assisted technologies in the writing process

During the preparation of this work, the authors used ChatGPT to improve grammar and enhance language clarity and efficiency. After using this tool, the authors carefully reviewed and edited the content as needed and take full responsibility for the content of the published article.

## Funding

None.

## Disclosures

None.
